# Nanocrystalline hexagonal diamond formed from glassy carbon

**DOI:** 10.1038/srep37232

**Published:** 2016-11-29

**Authors:** Thomas. B. Shiell, Dougal G. McCulloch, Jodie E. Bradby, Bianca Haberl, Reinhard Boehler, David. R. McKenzie

**Affiliations:** 1 Department of Electronic Materials Engineering, Research School of Physics and Engineering, The Australian National University, Canberra, ACT 2601, Australia; 2 School of Science, RMIT University, Melbourne, VIC 3001, Australia; 3 Chemical and Engineering Materials Division, Neutron Sciences Directorate, Oak Ridge National Laboratory, Oak Ridge, TN 37831, USA; 4 Geophysical Laboratory, Carnegie Institute of Washington, 5251 Branch Rd., NW Washington, DC 20015, USA; 5 School of Physics, The University of Sydney, NSW 2006, Australia

## Abstract

Carbon exhibits a large number of allotropes and its phase behaviour is still subject to significant uncertainty and intensive research. The hexagonal form of diamond, also known as lonsdaleite, was discovered in the Canyon Diablo meteorite where its formation was attributed to the extreme conditions experienced during the impact. However, it has recently been claimed that lonsdaleite does not exist as a well-defined material but is instead defective cubic diamond formed under high pressure and high temperature conditions. Here we report the synthesis of almost pure lonsdaleite in a diamond anvil cell at 100 GPa and 400 °C. The nanocrystalline material was recovered at ambient and analysed using diffraction and high resolution electron microscopy. We propose that the transformation is the result of intense radial plastic flow under compression in the diamond anvil cell, which lowers the energy barrier by “locking in” favourable stackings of graphene sheets. This strain induced transformation of the graphitic planes of the precursor to hexagonal diamond is supported by first principles calculations of transformation pathways and explains why the new phase is found in an annular region. Our findings establish that high purity lonsdaleite is readily formed under strain and hence does not require meteoritic impacts.

Lonsdaleite, named in honour of the crystallographer Kathleen Lonsdale, has been reported in terrestrial sediments and its presence is used as a signature for impacts on the earth’s surface of extra-terrestrial objects^1^,^2^,^3^. Bundy and Kasper were the first to report the formation of a sample in the laboratory by static compression that contained diffraction signatures of lonsdaleite from well-ordered graphitic precursors at temperatures above 1000 °C and pressures above 13 GPa^4^. Their material contained lonsdaleite microcrystals (of 0.1 μm diameter) with a preferred orientation in which the <100> direction of lonsdaleite was parallel to the <001> direction of the graphite precursor. Evidence for lonsdaleite has also been reported when powdered cubic diamond, graphite and C_60_ undergo static uniaxial compression^4^,^5^, hydrostatic pressure^6^, or shock synthesis^7^,^8^,^9^,^10^. Regions of the phase diagram where lonsdaleite has been reported as stable or metastable have been restricted to formation temperatures above 800 °C^6^,^11^. A report was made of a recoverable hexagonal phase after static compression of carbon nanotubes at room temperature and pressures of 75 GPa^12^. However, the results obtained from diffraction and Raman spectroscopy were not consistent with lonsdaleite^2^ and did not show the expected orientational relationship with precursor graphitic planes. Recently, it has been proposed that laboratory synthesis of pure lonsdaleite may be restricted to shock compression above 170 GPa and 6,000–7,000 K, replicating the conditions close to meteor impact sites^13^. To this day, there have been no reports in the literature specifically confirming a recoverable lonsdaleite phase from any static compression experiment unless the temperature exceeded 800 °C.

Recent reports have argued that all experimental evidence for lonsdaleite can be explained by a high content of stacking faults in cubic diamond^14^, raising doubts about the existence of this phase. Many solids consist of layers that can be stacked in various ways to give a structure with either cubic or hexagonal symmetry. For example, metals such as cobalt and covalently bonded crystals such as BN have layers that can adopt either an ABAB… stacking sequence leading to hexagonal symmetry, or an ABCABC… stacking sequence that leads to cubic symmetry. Cubic diamond can show faults in its stacking sequence that lead to diffraction peaks distinct from those of the perfect crystal^15^. Recent work has quantified the extent of hexagonal stacking in published lonsdaleite diffraction data and concluded that there is an experimental challenge to prepare samples beyond 60% hexagonality^16^. Such a challenge is worth addressing because lonsdaleite offers exceptional properties including extreme hardness, potentially exceeding that of cubic diamond^17^. Further increases in hardness may be possible if lonsdaleite could be prepared in nanocrystalline form, as has been reported for nanocrystalline diamond^18^. However, experimental measurements of the properties of lonsdaleite have been hampered by an inability to produce pure samples. A recent report has highlighted the role of shear strain in promoting phase transformations from disordered and amorphous structures^19^. Here we present a low temperature synthesis route that exploits this strain induced mechanism that offers an opportunity for enhancing the level of hexagonal stacking above the 60% threshold to the point where the existence of lonsdaleite as a distinct structural phase is beyond question.

## Results

**Figure 1:**
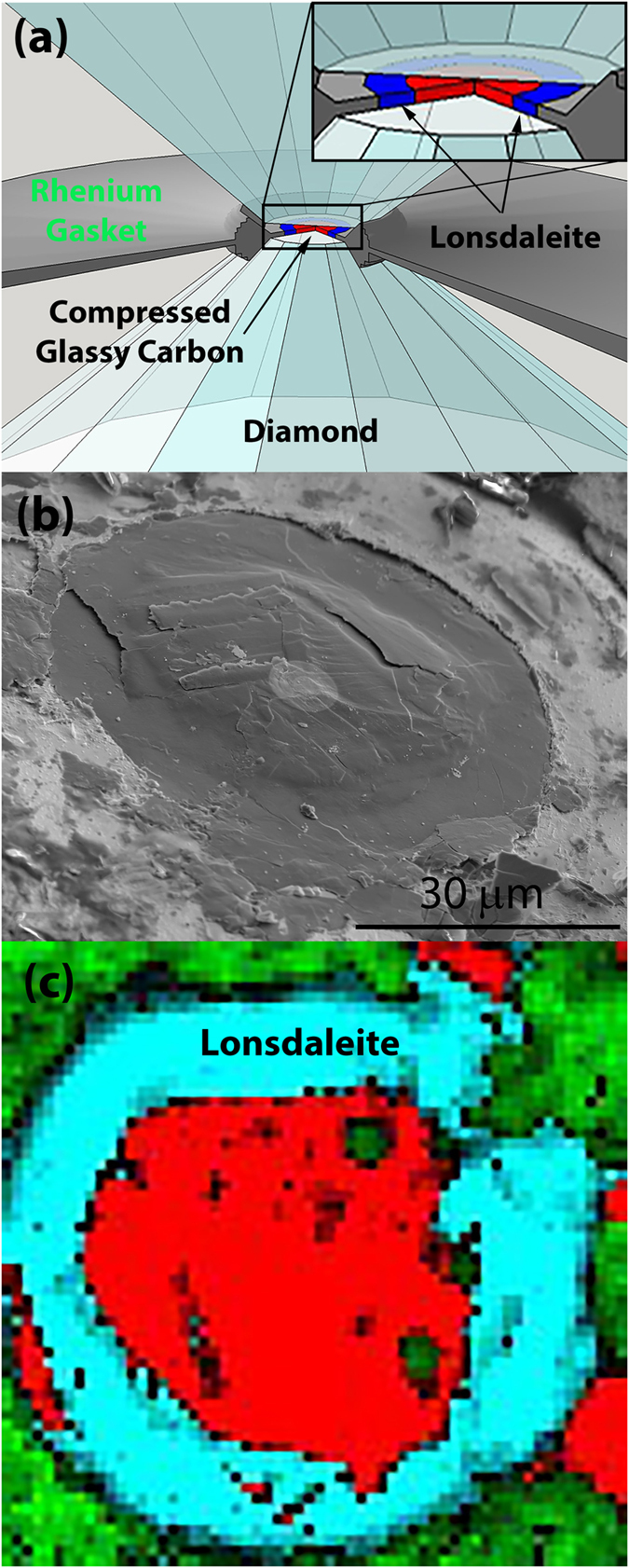
(**a**) Schematic view of the diamond anvil with the annular transformed region located between the diamonds. (Image courtesy of Larissa Huston.) (**b**) SEM image of the sample after recovery from the cell showing a domed central region (capped at the centre with a deposited platinum circle) with a denser annular region adjacent to the rhenium gasket. (**c**) Raman spectrum image showing the graphitic central region (red) and the annular region (blue) which had a distinctive Raman spectrum (see Supplementary Figure S2) resembling tetrahedrally bonded amorphous carbon^32^,^33^,^34^. The rhenium gasket is shown in green.

A glassy carbon specimen was placed in a diamond anvil cell and subjected to pressures up to 112 GPa at 400 °C for 2 hours (see Experimental Section for more details). Figure 1(a) shows a schematic of the diamond anvil cell, and Fig. 1(b) shows an SEM image of the sample after recovery from the cell. The strongly compressed annular region is transparent to infra-red light (see Supplementary Figure S1) while the thicker central region consists of opaque material. Raman spectroscopy (see Supplementary Figure S2) showed the annular region had a distinctive Raman spectrum with a single asymmetric peak resembling that of amorphous tetrahedrally bonded carbon^20^. The spectrum of the central region had graphitic features broadened from those of glassy carbon. The sharp boundary between the two regions in the Raman spectrum image shown in Fig. 1(c) is striking and represents the point where the conditions were conducive for a transition to a dense non-graphitic phase. Before discussing the detailed structure of the transparent annular region (that we show below to be lonsdaleite), the reasons for its annular geometry require comment. Recent modelling has shown that an annular transformed region can be formed in a material compressed between diamond anvils when the effect of strain is included^21^. The shear strain increases radially outwards from the centre of the sample as a result of the outward movement of material and facilitates the transition when the shear strain exceeds a threshold value. The sliding of the graphitic layers enables them to find optimal stacking^22^. Recent studies of the energetics for transformations from graphite related structures to diamond related structures show that the barriers for transformation to lonsdaleite are lower than to cubic diamond when the transformation proceeds stepwise^23^,^24^. Therefore, lonsdaleite will be preferred kinetically in situations where graphite layers are sliding over each other and small lonsdaleite crystals can be constructed by a progressive “locking in” of the favourable configurations as they occur. Moreover, finite element stress modelling of the diamond anvils has shown that cupping occurs at the high stress levels we used^25^. Any cupping of the diamond faces at high pressures^26^ would enhance the uniaxial compressive stress at the edge of the anvils, providing an additional driving force for the transition to a dense phase in an annular region.

**Figure 2:**
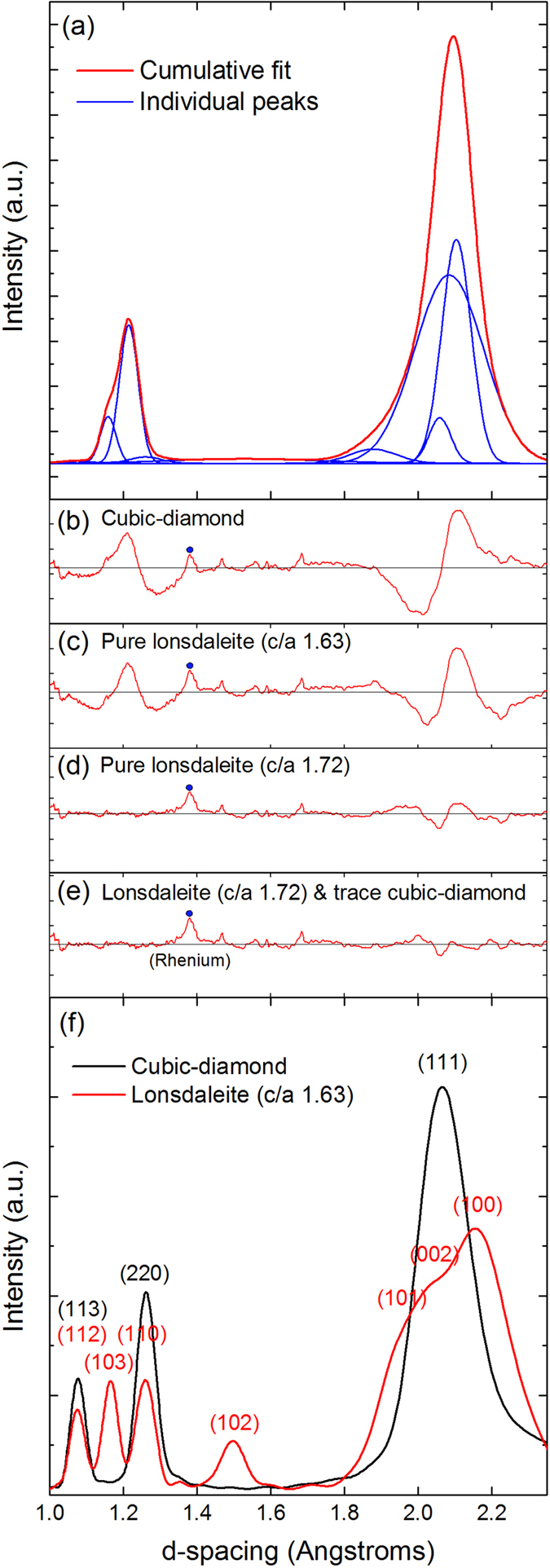
(**a**) The observed X-ray diffraction pattern showing the fitted peaks of lonsdaleite and cubic diamond. Details of fitted peak locations and intensities can be found in Supplementary Table 1 (See Experimental Section). (**b–e**) Residual plots of peak fitting attempts for different *a* and *c* lattice parameters. (**f**) Debye calculations of scattering intensity for clusters of 2000 atoms, with cubic diamond lattice parameter *a* = 3.5667^15^ and lonsdaleite lattice parameters of *a* = 2.52 and *c* = 4.12^4^. The major reflections have been indexed.

Using a focused high intensity beam at HPCAT at the Advanced Photon Source, Argonne National Laboratory, X-ray diffraction (XRD) was collected from the transparent annular region (see Fig. 2). The diffraction pattern could not be refined well with the cubic diamond structure or to lonsdaleite using the lattice parameters originally proposed, with a c/a ratio of 1.63^15^. However, our results were well refined with the lonsdaleite structure with unit cell parameters of a = 2.43 ± 0.005 Å and c = 4.17 ± 0.005 Å (see Supplementary Table 1 for details), giving a c/a ratio of 1.72. This c/a ratio is consistent with other experimental findings, as shown in Supplementary Table 2. It is likely that our observed departure from the ideal lonsdaleite ratio of 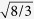 is caused by the shear driven formation mechanism. A slightly improved refinement was obtained by adding small peaks for cubic diamond, indicating that a trace of the cubic phase is also present. Comparing the intensity of the {111} diamond peak with the {002} lonsdaleite peak and weighting them with the expected intensity calculated for ideal cubic diamond and lonsdaleite using the Debye scattering formula [see Fig. 2(f)], our calculated fraction of lonsdaleite (100% hexagonal stacking) is 90 ± 5%. This fraction is significantly higher than all previous reports where lonsdaleite was identified^16^. While other polytypes of diamond in addition to the cubic phase may also be present, their lower degree of hexagonality compared to lonsdaleite^27^ limits them to trace amounts.

**Figure 3:**
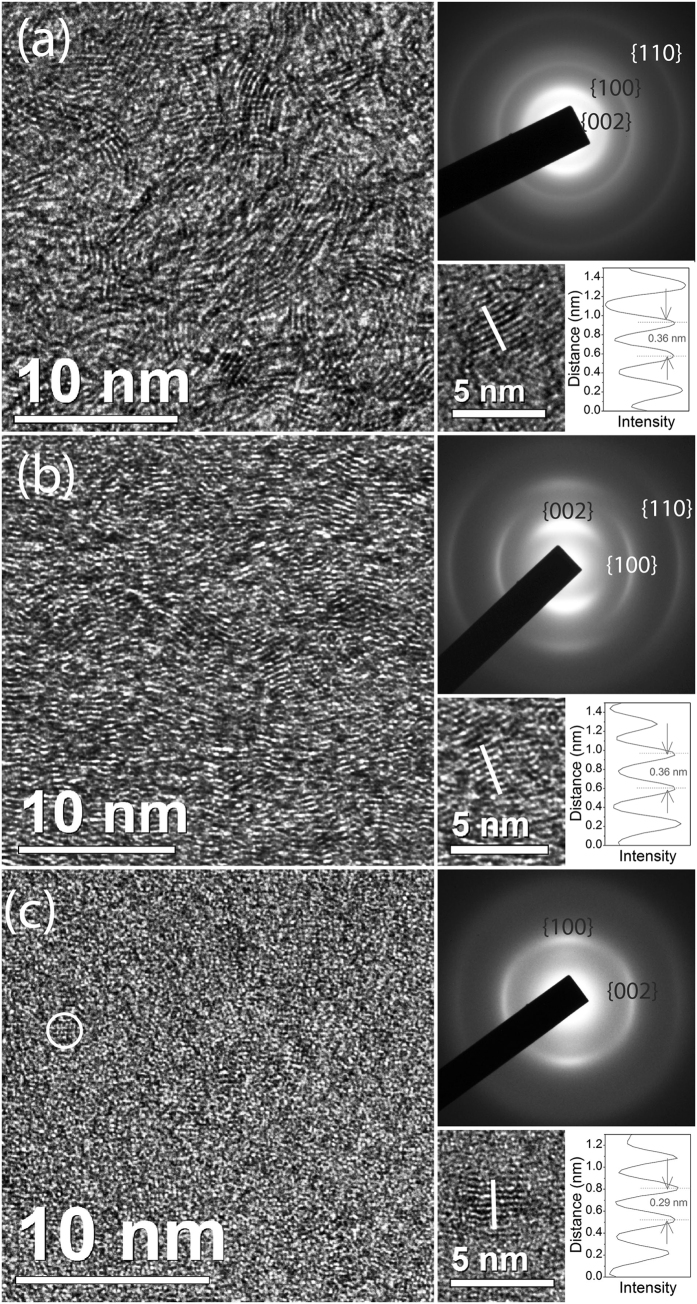
(**a**) TEM images and diffraction pattern (indexed to graphite) of the tangled graphitic ribbon-like microstructure of the glassy carbon precursor. (**b**) TEM images and a diffraction pattern (indexed to graphite) of the central region of the recovered specimen subjected to high pressures (with the stress direction aligned vertically). (**c**) TEM images and a diffraction pattern (indexed to lonsdaleite) of the annular region of the recovered specimen (stress direction aligned vertically). A lonsdaleite crystal has been circled in part (**c**) with a diameter of 1–2 nm. Also shown on the bottom right of each panel are examples of graphitic inclusions. The graphitic interplanar distance has been compressed to 2.9 Å in the annular region.

A focussed ion beam was used to extract transmission electron microscope (TEM) lamellar specimens from the precursor glassy carbon and from the central and annular regions (see Supplementary Figure S3). The TEM image and diffraction pattern of the precursor glassy carbon [Fig. 3(a)] reveals a tangled graphitic microstructure characteristic of this material^28^. The TEM image of the central region [Fig. 1(b)] shows highly oriented graphitic layers arranged normal to the uniaxial stress direction [Fig. 3(b)]. The graphitic {002} reflections, aligned with the stress direction in the diffraction pattern [Fig. 3(b)], confirm the strong preferred orientation and reveal an interplanar spacing similar to that of graphite. The TEM image of the annular region [Fig. 3(c)] mainly shows small crystallites ~2 nm in size (as indicated in the figure). Also in the annular region are occasional graphitic inclusions aligned with layers normal to the stress direction [bottom right of Fig. 3(c)]. The interplanar spacing of these areas was measured to be ~2.9 Å, smaller than for ordinary graphite. Trace amounts of compressed graphite have previously been observed in association with lonsdaleite^4^. The diffraction pattern from the annular region [Fig. 3(c)] has been indexed to lonsdaleite. As expected, the direction of the uniaxial component of the stress is parallel to <100> in lonsdaleite^4^. This is the low barrier pathway from compressed graphite to lonsdaleite expected from modelling studies^24^. The crystals of lonsdaleite do not form in the central region because the shear strain is insufficient to enable growth of energetically favourable stacking.

Electron energy loss spectroscopy (EELS) provides information on density and chemical bonding of the carbon atoms^29^ and was performed on the precursor glassy carbon, the central, and the annular regions of the recovered sample (see Supplementary Figure S4). The bulk plasmon peak position, an indicator of density, collected from the central region was found to occur at 27 eV compared to 22 eV in the precursor. This increase in peak position indicates that the central region has been significantly densified. The annular region shows a spectrum similar to cubic diamond^6^,^30^ with a plasmon peak at ~32 eV, correlating to a density of 3.3 g/cm^3^, which is consistent with nanocrystalline lonsdaleite containing a minority of compressed graphite. The carbon K-edge fine structure, which is dependent on local bonding, taken from the annular region (see Supplementary Figure S4) is dissimilar to cubic diamond, but is similar to that reported by Sato *et al.*^30^ for lonsdaleite.

**Figure 4:**
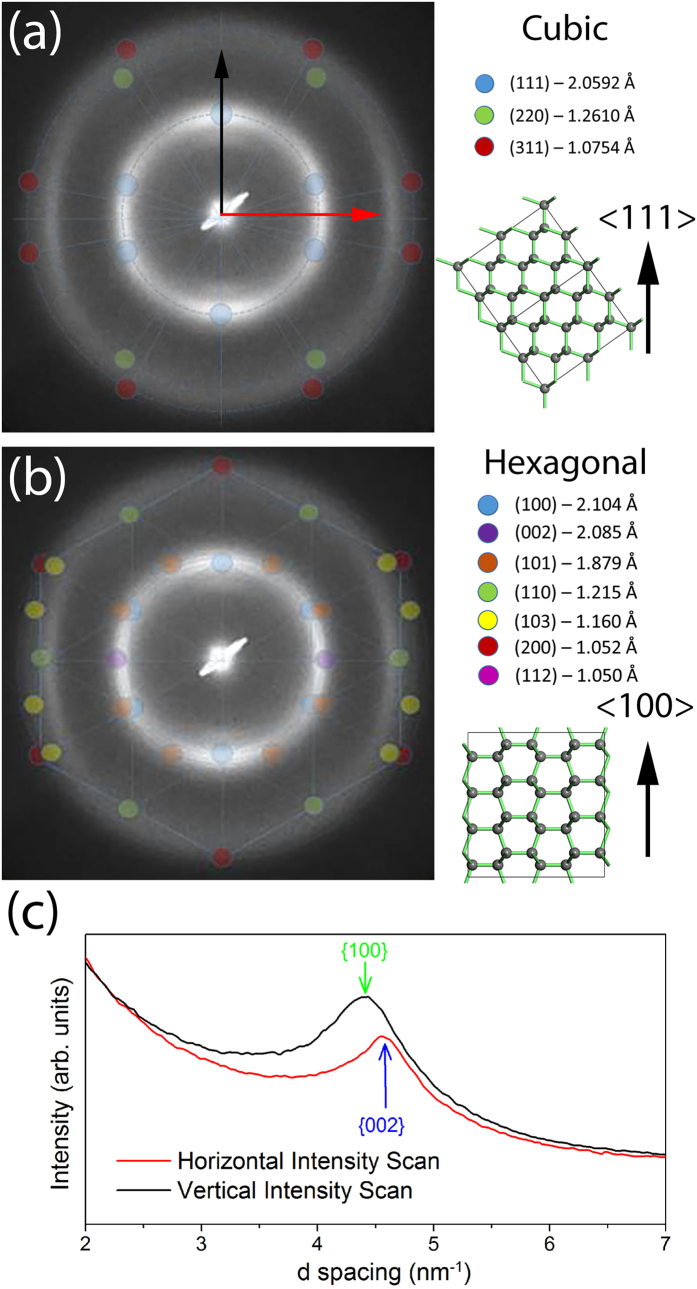
(**a**) The electron diffraction pattern (aligned so that the stress direction is vertical) from the annular region of the recovered sample compared with the predicted reflections according to polycrystalline cubic diamond with the <111> direction vertical. The spots indicate the intersection of the Ewald sphere with the reciprocal lattice freely rotating around <111>. (**b**) The diffraction pattern compared to the corresponding prediction for polycrystalline lonsdaleite with the <100> direction vertical. The lonsdaleite mode matches the key features including: unequal diameters of the vertical and horizontal peaks on the inner ring due to the different {100} and {002} d-spacings [shown more clearly in the intensity linescans in (**c**)] and the correction prediction of the horizontal second ring diameter arising from the {110} reflections of lonsdaleite.

To explain the preferred orientation of lonsdaleite crystallites in the annular region [Fig. 3(c)], the electron diffraction pattern viewed normal to the stress direction was analysed assuming the sample contained nanocrystalline lonsdaleite and nanocrystalline cubic diamond. These nanocrystalline models assume a random orientation of the crystallites around a unique axis, aligned with the stress direction. For lonsdaleite, this axis is the <100> direction while for cubic diamond it is the <111> direction, as shown in Fig. 4. For each model, the reciprocal lattice is rotated about the unique axis and then intersections of lattice points with the Ewald sphere are indicated in Fig. 4(a) for cubic diamond and Fig. 4(b) for lonsdaleite. The reciprocal lattice points become arcs because of a distribution of angles of the crystallites around the unique axis. The lonsdaleite model matches the key features of the observed diffraction pattern including the unequal diameters of the arcs on the inner ring in the vertical and horizontal directions [due to the different {100} and {002} d-spacings, as shown more clearly in Fig. 4(c)], the presence of the {110} arc at right angles to the stress direction, and the correct angular distribution of intensity around the first and second diffraction rings. The electron microscopy analysis yielded a c/a ratio of 1.71, in line with our XRD refinement.

## Discussion

We now discuss the pathways to formation of this form of nanocrystalline lonsdaleite. A pathway that requires the sudden transformation of the entire structure necessitates the coherent motion of large numbers of atoms. While this is a possibility for a crystalline precursor, our non-crystalline precursor with imperfect layer orientation is not amenable to such a pathway because the initial positions of the atoms are not correlated. Khaliullin *et al.* have proposed that the transformation from hexagonal graphite to lonsdaleite, occurs most readily by a step by step mechanism in which layers are added one by one, rather than a concerted mechanism that involves bulk reconstruction in which many layers move coherently^23^. Xiao and Henkelman have calculated the lowest energy barriers for the transformation by nucleation and growth of the hexagonal graphite to lonsdaleite transition^24^. They have found that the barrier for a kinetically driven, layer by layer transformation is lower than the transformation of rhombohedral graphite to cubic diamond although the energy of the cubic transformed state is slightly lower as it is the thermodynamically preferred structure^24^. In the strain induced transformation scenario we propose, the formation of hexagonal stacking will proceed as graphene layers slide over each other during the intense radial plastic flow in the material between the anvils^21^. Progressive “locking in” of energetically favourable structures will build small lonsdaleite crystals. The growth will stop when progress in propagation of the crystallisation is too slow. This happens after relatively few layers have achieved hexagonal transformation since the precursor has only poorly ordered layers that achieve coherent registration over small distances. A nanocrystalline structure is the result. The crystals of lonsdaleite do not form in the central region because the plastic flow is insufficient to enable energetically favourable hexagonal stacking to occur frequently enough. Well-ordered graphitic precursors undergoing the same experimental procedure would likely result in larger sized lonsdaleite crystals. Indeed, the strain induced synthesis route has recently been proposed in the formation of wurtzitic boron nitride which is an analogue for lonsdaleite in the carbon system^19^.

In summary, we show that a stable, recoverable transparent nanocrystalline lonsdaleite with the expected orientation relationship to precursor graphitic planes is produced by the shear strain of an intense radial plastic flow under static compression. The low energy barrier for a progressive transformation from graphitic layers to lonsdaleite is proposed as the underlying cause of this kinetically driven process occurring at moderate temperatures well below those previously reported for lonsdaleite. The material we have produced has an overwhelming predominance of the ABAB… stacking sequence, qualifying it as lonsdaleite rather than defective cubic diamond. The results of this study highlight the importance of shear strain as a powerful mechanism to induce phase transformations in carbon that would otherwise require high temperatures.

## Methods Section

### Precursor material and sample preparation

The glassy carbon precursor used in this study, denoted Sigradur-G (purchased from Hochtemperatur-Werkstoffe), had undergone an initial heat-temperature-treatment at 3000 °C and has a density of 1.42 g. cm^−3^ (as supplied by the manufacturer). A 50 × 50 × 20-μm shard of this material was mounted into the sample chamber of a Boehler plate diamond-anvil-cell (DAC) that had 120 μm diameter culets and a pre-indented and drilled Rhenium gasket with a thickness of 15–20 μm and a hole of diameter 60 μm. No pressure-medium was used. Pressure inside the DAC was increased incrementally up to a maximum of 112 GPa, where it was annealed at 400 °C for 2 hours in an oven. Rhenium diffraction peaks were used for pressure determination.

### Infrared transmission measurements

All images were taken on a Motic BA310Met-T microscope with a 3.1 MP industrial digital camera (manufactured by ToupTek Photonics). The microscope was equipped with a 50x objective lens (WD = 8.4 mm, NA = 0.45). The sample was illuminated by a 50 W halogen light source and the exposure time was set to 2 s.

### Raman spectroscopy measurements

All Raman spectra were taken on a Renishaw InVia micro-Raman Spectrometer. A 50 mW 532 nm diode laser was used for excitation. The spectrometer was equipped with a with a Nikon 50x objective lens (WD = 17 mm, NA = 0.45), which produced a focal spot of 1 μm^2^ and a total power of 0.71 mW from the objective. All spectra were processed to remove cosmic rays using the inbuilt software package Wire 4.1.

### X-ray diffraction measurements

All X-ray diffraction (XRD) measurements of the initial precursor glassy carbon and the hexagonal-diamond sample were conducted at 16-ID-B beamline of HPCAT, at the Advanced Photon Source. This facility provides a collimated monochromatic X-ray beam with a beam energy of 30 keV and a FWHM of approximately 5 × 7 μm. All scans ran for a minimum of 30 seconds on a 1 M Pilatus detector to ensure an acceptably low noise-to-signal ratio. All raw data was initially processed using Dioptas 2.3^31^ to remove features from the apparatus. All XRD background removal and peak fitting for refinement was conducted manually using OriginPro 9.1.

### Transmission-electron-microscopy measurements

Several lamellae were cut from the surface of the sample and from a piece of unpressurised, non-annealed glassy carbon using a FEI Scios dual beam FIB and an associated *in-situ* plucking technique (see Supplementary Figure S3). All bright-field TEM images, electron diffraction patterns, and EELS measurements were taken using a JEOL 2100 F TEM (RMIT University, Australia), and were subsequently processed using Gatan Digital Micrograph software.

## Additional Information

**How to cite this article**: Shiell, T. B. *et al.* Nanocrystalline hexagonal diamond formed from glassy carbon. *Sci. Rep.* **6**, 37232; doi: 10.1038/srep37232 (2016).

**Publisher’s note:** Springer Nature remains neutral with regard to jurisdictional claims in published maps and institutional affiliations.

## Supplementary Material

Supplementary Information
