# Supplementary material for: Nanocrystalline hexagonal diamond formed from glassy carbon
Source: Sci Rep. 2016 Nov 29;6:37232. doi: 10.1038/srep37232 (PMC5126635; doi:10.1038/srep37232)
Supplement: Supplementary Information [file srep37232-s1.pdf]

Title: Nanocrystalline hexagonal diamond formed from glassy carbon

Authors:
Thomas. B. Shiell[1]
Professor Dougal G. McCulloch[2]
Associate Professor Jodie E. Bradby[1]
Doctor Bianca Haberl[3]
Doctor Reinhard Boehler[4]
Professor David. R. McKenzie[5] * (Corresponding author, email: David.McKenzie@Sydney.edu.au)

[1]Department of Electronic Materials Engineering, Research School of Physics and Engineering, The Australian National University, Canberra, ACT 2601, Australia
[2]School of Applied Sciences, RMIT University, Melbourne, VIC 3001, Australia
[3]Chemical and Engineering Materials Division, Oak Ridge National Laboratory, Oak Ridge, TN 37831, USA
[4]Geophysical Laboratory, Carnegie Institute of Washington, 5251 Branch Rd., NW Washington, DC 20015, USA
[5]School of Physics, The University of Sydney, NSW 2006, Australia

Supplementary Figures:

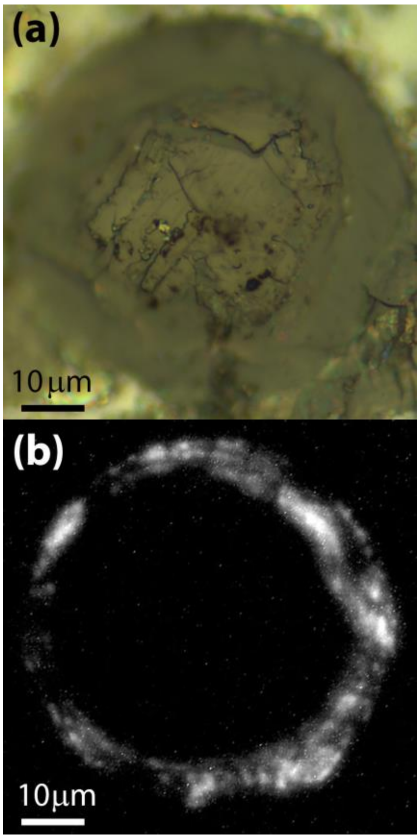

Supplementary Figure S1: (a) An optical image of the carbon sample after extraction from the diamond anvil cell. (b) An infrared transmission image showing the transparency of the annular region.

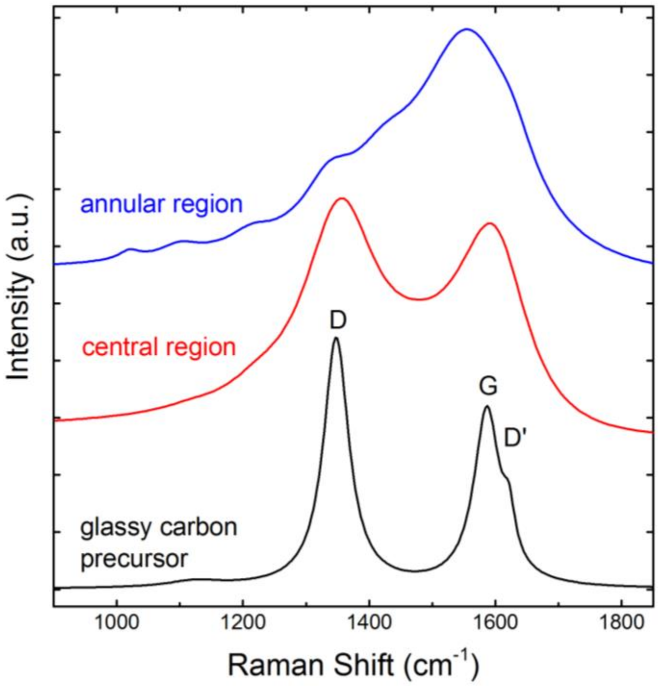

Supplementary Figure S2: Raman spectra of the central and annular regions. Also shown for comparison is the spectrum from the glassy carbon precursor (black). Clearly defined D, G and D'-peaks can be seen in a Raman spectrum of the GC precursor. [32] The D and G-peaks of the central region (red) have become quite convoluted, indicative of an increase in disorder. Raman spectrum of the annular region is shown in blue and is consistent with spectra previously reported from CVD-grown nano-diamond. [32-34]

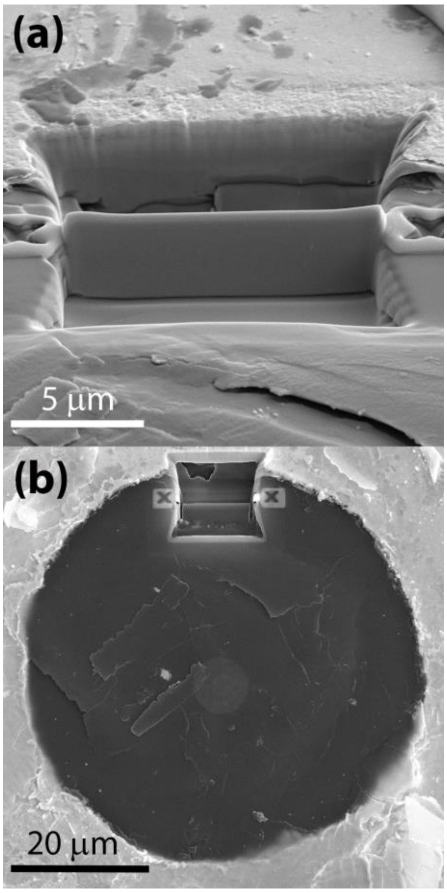

Supplementary Figure S3: (a) SEM image of a single lamella extraction. (b) SEM image showing the location of the lamella from part (a) cut out of the annular region for TEM analysis. Several lamellae were subsequently cut from both the central and annular regions.

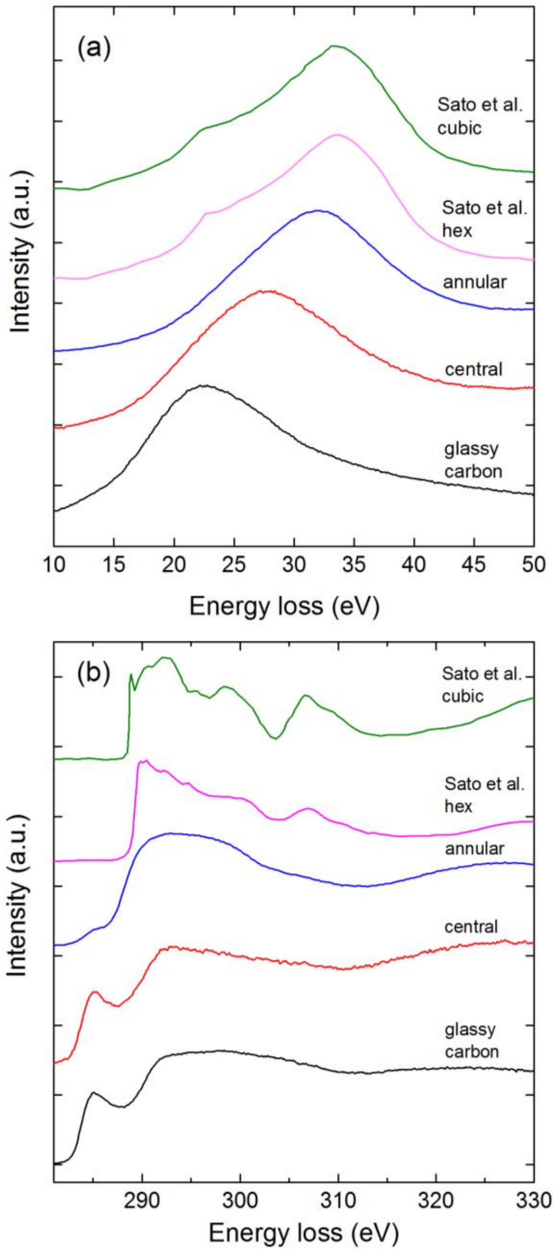

Supplementary Figure S4: EELS spectra taken from the annular, central and precursor material. (a) Low-loss EELS spectra of the precursor material, and of the central and annular regions of the sample showing the increase in overall density after the high temperature high pressure treatment. (b) K-edge spectra exhibiting a substantial peak at 285 eV for both the central region and the precursor material, but not for the annular region. This indicates a substantial drop in the abundance of sp^2 bonding present in the annular region. For comparison the corresponding low-loss and K-edge spectra from previously published cubic and hexagonal diamond are shown in (a) and (b). [30]

Supplementary Tables:

	[hkl]	d-spacing$_{(calc)}$ (Å)	Intensity$_{(obs)}$
Hexagonal-diamond	100	2.104	medium
	002	2.085	strong
	101	1.879	weak
	110	1.215	medium
	103	1.160	weak
Cubic-diamond	111	2.059	weak
	022	1.261	very weak
	113	1.075	very weak
Graphite	002	3.35 – 3.5	Out of range
	100	2.1	DNE
	110	1.23	DNE

Supplementary Table 1: Details of the peaks fitted to the X-ray diffraction intensity data shown in Supplementary Fig. 3(a).

	a (Å)	***c*** (Å)	***c/a***
This Work (X-rays)	2.43	4.17	1.72
Kraus [13]	-	-	1.69
Yoshiasa [11]	2.508	4.183	1.67
Wang [12]	2.496	4.123	1.65
Bundy [4]	2.52	4.12	1.63

Supplementary Table 2: Lattice parameters *a* and *c* and the ratio *c/a* determined in this work compared to the previously published values calculated from XRD measurements.
